# 

**Published:** 1848

**Authors:** 


WHOLE SERIES—VOL. V, NO. I.

VOL. I.] APRIL AND MAY. [No. 1.

### THE NORTH-WESTERN

Dr J. B. Jonsman

### MEDICAL AND SURGICAL JOURNAL.

PUBLISHED EVERY OTHER MONTH,

TWO DOLLARS PER ANNUM, IN ADVANCE.

EDITED BY
WILLIAM B. HERRICK, M.D., AND JOHN EVANS, M.D.,
PROFESSORS IN RUSH MEDICAL COLLEGE, CHICAGO.

CHICAGO AND INDIANAPOLIS:
PUBLISHED BY JOHN W. DUZAN.
INDIANA STATE JOURNAL—PRINT.
1848.

ENLARGED SERIES—VOL. III, NO. I.

